# Perspective: Achieving Sustainable Healthy Diets Through Formulation and Processing of Foods

**DOI:** 10.1093/cdn/nzac089

**Published:** 2022-04-30

**Authors:** Adam Drewnowski, Patrick Detzel, Petra Klassen-Wigger

**Affiliations:** Center for Public Health Nutrition, University of Washington, Seattle, WA, USA; Novartis, Basel, Switzerland; Nestlé Research and Development, Vers-chez-les-Blanc, Lausanne, Switzerland

**Keywords:** formulation, food processing, fat, sugar, salt, fortification, micronutrients, nutrient profiling, regulation

## Abstract

Food processing and food (re)formulation can contribute to achieving sustainable healthy diets. Distinct from product formulation, the main purpose of food processing is to provide a stable and resilient supply of safe, shelf-stable, and affordable foods. Although efforts at reformulating processed foods have focused on removing excess added fat, sugar, and salt, product formulation can also take the form of voluntary fortification with protein, fiber, and micronutrients to improve dietary nutrient density and address population health needs. Advances in food technology have also led to the addition of desirable ingredients, including plant-based proteins and fermentation products, to processed foods. Among continuing challenges to product (re)formulation are the need to ensure product safety, maintain sensory appeal, control product cost, assure consumer acceptance, and manage the environmental footprint across the value chain. Voluntary (re)formulation of processed foods by the food industry can help improve diet quality and food security for all.

## Teaser

Food processing and food (re)formulation are distinct concepts. Formulation and reformulation of processed foods can improve nutrient density and healthfulness of the food supply at an affordable cost.

## Introduction

Guiding principles for achieving sustainable healthy diets were recently developed by the FAO and the WHO (1). Sustainable healthy diets need to be healthy and safe, economically affordable, socioculturally acceptable, and with low environmental impact (1, 2). The affordability of healthy diets in the context of food security was the topic of another FAO report (3). Based on FAO estimates (4), the cost of healthy diets was far in excess of the international poverty line (US$1.90/d). One question with implications for global public health nutrition is whether the sought-after affordable nutrient density for all (3) can be achieved without processed foods.

Although the promotion of fresh unprocessed foods remains the primary health goal (5), experts do acknowledge that a parallel reformulation of processed foods would improve population diets. The WHO has encouraged the private sector to produce more food products that eliminate *trans* fats, reduce saturated fat, and are lower in sugar and salt (6). Industry policies, research, and development need to encourage the production of lower cost and more nutrient-dense foods.

Food processing and food (re)formulation are distinct concepts. The role of food processing is to transform perishable agricultural products into foods and beverages that people consume (7). Food processing contributes to the stability of the food supply, helps minimize price spikes and price volatility, and allows food systems to withstand emergencies, disasters, and shocks (8). Though product reformulation has become identified with reducing fat, sugar, and salt, it can also include enrichment or food fortification with vitamins and minerals (9). Many low- and middle-income countries (LMIC) still face the problem of inadequate nutrients and the frequent lack of high-quality protein (10). Some challenges to product reformulation are discussed below.

## Product Reformulation

Four approaches to product reformulation are identified in this report and summarized in **Table 1**. The first involves total removal of industrial *trans* fat and partial or total removal of added fat, added sugar, and sodium. The second involves mandatory or voluntary product fortification with protein, fiber, vitamins, and minerals. The third involves the addition to processed foods of minimally processed desirable components (whole grains, fruit, nuts, seeds). The fourth involves technological innovation and the use of new functional ingredients to create a new generation of processed plant-based foods.

**TABLE 1:** Selected aspects of product reformulation for health

	Remove or reduce	Add
Energy and nutrients	Energy, fat, trans fat, sugar, sodium	Protein, fiber
Micronutrients	Antinutrients, phenols	Calcium, iron, zinc, iodine, folate, vitamins A, D, B-12
Ingredients		Whole grains, fruit, nuts, seeds
Functional ingredients		Plant protein isolates, sweeteners, salt alternatives

### Reformulation by reducing fat, sugar, and salt

The processing of food is ancient and necessary. The goal is to make food safer, tastier, and more nutritious, and to reduce or avoid food spoilage, loss, and waste (11). Fat, sugar, and salt were initially added to foods for preservation, safety, and storage (11). Traditional means of preserving perishable foods used hyperosmotic solutions—either sugar syrup or brine—to remove water from the fresh food matrix. Water could also be removed from fresh foods through drying and air curing. These traditional methods are still being used by small entrepreneurs and at home (12). However, their role has diminished with the availability of cold storage.

There are several technical options to reduce or remove fat, sugar, and salt from processed foods. It is important to note that these ingredients have important functional properties, helping to maintain food moisture, and providing mouthfeel, volume, and bulk (13). The first option is to reduce the amount of the target ingredient without replacing it. However, skim milk, beverages with reduced sugar content, and lower-sodium bread, pizza, and soups might not have the same sensory qualities as the original product (13, 14). The second option is to use substitutes for fat, sugar, and salt. These substitutes can include starches, gels, and gums, low-calorie sweeteners, potassium chloride and glutamate salts, along with a variety of herbs and spices.

The third option is to add a lower-calorie bulking agent. Carbohydrates, proteins, emulsifiers, gums, and gels have substituted for fat, providing the necessary mouthfeel, viscosity, and volume (13). The fourth option relies on advanced technology to mimic sensory qualities of fat, sugar, or salt while minimizing the amount. Examples are nonabsorbable fats, altered salt crystals, and hollow sugar crystals (15). Successful product reformulation is subject to technical constraints and requires continued consumer acceptance.

### Reformulation through mandatory or voluntary fortification

Fortification strategies to improve nutrient density of foods are guided by the FDA in the United States (16, 17). Manufacturers can add essential nutrients to a processed food to correct a dietary insufficiency at the population level or to meet a demonstrated public health need. The target foods need to be affordable, eaten regularly, and in predictable amounts. The added nutrients must remain stable and bioavailable and must not alter the appearance or the sensory qualities of food. As listed in the Global Fortification Data Exchange (18), 139 countries have fortification standards for selected food vehicles. Typical targets for mandatory fortification (enrichment) in lower income countries are starches and fats, including wheat flour, cornmeal, noodles, and cooking oils, margarine, and ghee (19). Mandatory fortification of salt with iodine has had major public health impact across the LMIC (20). Voluntary fortification can apply to a wide range of micronutrients, including B-vitamins, iron, and zinc, depending on the specific nutritional needs of the target population (19, 20). For example, sugar, snacks, beverages, and candy have been among the fortification vehicles used in LMIC. Sugar has been used as a mandatory vehicle for vitamin A fortification in Guatemala, El Salvador, and Honduras (21). Other examples include iodized and doubly fortified salt (20) and quadruply fortified salt (22). Doubly fortified salt contains iron and iodine; quadruply fortified salt contains iodine, iron, folic acid, and vitamin B-12 (22). Bouillon cubes are a prominent vehicle for voluntary fortification (23, 24).

### Reformulation through technology advances

Another type of formulation involves adding desirable ingredients (pulses, nuts, seeds, whole grain) to processed foods. Technological advances have also allowed the development of plant-based alternatives to meat, fish, and dairy proteins that are, at the same time, tasty, nutrient dense, affordable, and have a lower environmental footprint (15). Their production requires attention to variety, selection, and the breeding of plants for optimal taste, yield, and nutritional quality (15). Plant-based alternatives to animal source foods are formulated for optimal nutrient density, low fat and sugar content, and adequate content of essential micronutrients.

## Drivers and Challenges to Reformulation

### Benchmarking reformulation using nutrient profiling methods

Initially developed to assist in the regulation of nutrition and health claims in the European Union, nutrient profiling (NP) models have served to regulate marketing and advertising to children and have provided the scientific basis behind front-of-pack nutrition labels and logos (25, 26). Most were designed by governments and nongovernmental organizations to shift food purchases toward healthier foods (25). However, the impact of NP-driven food labels or logos on consumer food choices appears to have been relatively small (27).

By contrast, NP models have had a profound effect on product reformulation efforts by the food industry (28). Many food companies have developed their own NP systems to screen nutrient density of product portfolios, benchmark nutrition targets, and guide product reformulation (29). What is more, food and beverage companies are increasingly being judged based on reformulation efforts and on the nutrient density of product portfolios. The UK Product Profile 2021 (30), published by the influential Access to Nutrition Initiative (ATNI), used the Health Star Rating to screen >4000 products from 16 large food companies that accounted for ∼50% of total UK retail sales in 2019. The ATNI Global Index 2021 (31) commends food companies for developing NP systems, using such systems to screen product portfolios, and making NP standards publicly available. A recent ATNI report noted that 13 of 22 large food companies used NP methods to improve nutritional quality of their products (31). Industry-developed NP methods have become a driving force for innovation and product reformulation.

### Reformulation health outcomes

Mandatory sodium reduction in the United Kingdom is cited as the greatest public health success (32), after *trans* fats. Among the UK foods affected by mandatory sodium reduction targets were bacon, sausages, meat pies, bread, rolls and “morning goods,” breakfast cereals, cheese, salted butter, fat spreads, prepared meals, soups, pizzas, crisps and snacks, sandwiches, cakes, sauces, puddings, pasta, cereals, canned foods, and meat alternatives. Sodium in many foods was reduced by ≤40–50%, and >11 million kg of salt was removed from the UK food supply. The observed reduction in sodium intakes was credited to product reformulation and not to any change in consumer behavior, exemplifying the covert “health by stealth” approach. Still, average salt consumption in the United Kingdom remains high at 8.1–8.8 g/d and well above the target goal of 6 g/d for adults.

However, there is little observational evidence so far that reducing sodium had a significant impact on product sales or on population health. Sales of reformulated products can depend on the type of products, prices, length of transition period, and any associated public health campaigns. Much of the evidence on the impact of reformulation has come from modeling studies, systematic reviews, and meta-analyses (33). Among frequent modeling approaches were post facto analysis of product reformulation campaigns and the likely impact of product reformulation on health outcomes (33). Systematic reviews, mostly of the sodium reduction literature, point to a small positive health impact overall (33). By contrast, product reformulation data from LMIC generally show that mandatory or voluntary fortification of foods with essential nutrients has reduced prevalent undernutrition and nutrient deficiencies in a cost-effective way.

The covert “stealth reformulation” approach has been criticized on the grounds that it does not involve drastic dietary change (34). The concerns are that product reformulation would endorse, legitimize, or even promote the consumption of processed and “ultra-processed” foods (34). That would undermine existing public health policies to promote the consumption of fresh, unprocessed and minimally processed foods (5). However, as noted in recent FAO reports (3) and other studies (4), fresh unprocessed foods generally cost more.

Taxation was another indirect approach to encourage sugar reduction in beverages and foods (35). Seventeen countries, including 13 Organization for Economic Co-operation and Development (OECD) countries, tax sugar-sweetened beverages and selected other foods (35). Some examples of taxation policies include “soda taxes” in the United Kingdom, France, Mexico, Chile, and places in the United States including the cities of Berkeley and Seattle. There is some evidence from the United Kingdom that the soft drink tax has led to product reformulation (36). Although some changes in consumer purchases were observed (from higher sugar to lower sugar beverages), an estimated 73% of the decrease in total sugar consumption came from reformulating existing products or the introduction of new ones.

### Challenges to product reformulation

Reformulating an existing food product is not always technically simple. There are complex interactions among food technology limitations, nutrition economics, and consumer behavior. Among the many technical challenges are preserving sensory qualities of the product, controlling cost, and assuring product safety. There can also be regulatory challenges related to standards of identity and local regulations on enrichment, fortification, advertising, and marketing. Product reformulation by small and medium-size enterprises can have its own set of challenges.

Issues of taste and cost are often critical. When it comes to consumer acceptance, nutrient density is not the only factor that matters. Taste, cost, and convenience matter more. Consumers might not accept an altered product that is healthier but tastes worse and costs more. The reformulated products might not have the same sweetness, texture, or mouthfeel, and the replacement of sugar might not be perceived as “natural.”

Product reformulation also needs to conform to mandatory standards of identity, where appropriate. In the United States, FDA standards of identity describe what a food product must contain, in what proportions, and sometimes how it must be manufactured. For example, based on standards of identity, chocolate with sharply reduced cacao fat cannot be called chocolate: it is a chocolate product. Standards of identity do not yet apply to plant-based “milks” in the United States.

Second, the provision of affordable nutrient density to the consumer is of critical importance to health equity and global public health. The FAO report on affordable nutrition noted that healthier diets were often not affordable to lower-income groups (3). That much is acknowledged in the OECD report (35, 36), which is one of the few to note that product reformulation is not always a part of the normal “process” and does involve additional cost—from technology development to marketing and consumer outreach. Reformulation requires an investment in research, machinery, and other production processes and can change production cost. This can have an impact on product price and on sales.

## Toward Affordable Nutrient Density in Processed Foods

Food reformulation is distinct from food processing that provides safety, convenience, reduced consumer food waste, and affordable cost. A recent article on the processed food revolution in Africa (37) emphasizes that purchases of processed food are driven by long-term factors, such as urbanization, increased income, and employment changes, noting that policies cannot change the pursuit of convenience and labor-saving food.

Second, processed foods ensure safety, prolong shelf life, and minimize food loss and waste at consumer level. Third, fortified processed foods provide affordable nutrient density, help minimize disparities in access to food, and ensure nutrition security for all. Contrary to the notion that all processed foods are unhealthy, Reardon et al. (37) note that much processed food, like packaged milk, supports sound nutrition, and that the processed food system is a major source of jobs for women.

Within the context of global food systems, it is important to address the positive aspects of reformulation. New food technologies offer multiple opportunities for improving global public health. Nutrient density of foods can be improved by reducing energy from sugar and fat and by simultaneously increasing the content of vitamins, minerals, and high-quality protein. Desirable food ingredients—fruit, legumes, dairy, and nuts and seeds—can also be incorporated in processed foods at an affordable cost.

The food industry needs to engage with public health agencies and with the consumer to improve the quality of processed foods. Following FAO principles, sustainable healthy diets need to be affordable, adapted to regional and local food habits, and consistent with planetary boundaries. Reformulation of processed foods can contribute to food security for all.

## Notes

The present analyses were supported by Nestlé.

Author disclosures: AD is a member of the Nestlé Scientific Advisory Board. AD has received grants, contracts, and honoraria from entities both public and private, with an interest in dietary nutrient density and nutrient profiling of foods. PK-W is with Nestlé Research and Development. PD was with Nestlé at the time of this work and is now with Novartis.

Abbreviations used: ATNI, Access to Nutrition Initiative; LMIC, low- and middle-income countries; NP, nutrient profiling; OECD, Organization for Economic Co-operation and Development.

## References

[1] FAO, WHO . Sustainable healthy diets: guiding principles [Internet]. 2019; [cited April 25, 2021]. Available from: http://www.fao.org/3/ca6640en/ca6640en.pdf.

[2] HLPE . Nutrition and food systems: a report by the High Level Panel of Experts on Food Security and Nutrition. [Internet]. Rome: Committe on World Food Security, FAO; 2017; [cited April 25, 2021]. Available from: https://www.fao.org/3/i7846e/i7846e.pdf.

[3] FAO, IFAD, UNICEF, WFP, WHO . The state of food security and nutrition in the world: transforming food systems for affordable healthy diets [Internet]. Rome: FAO; 2020; [cited April 25, 2021]. Available from: https://www.fao.org/3/ca9692en/online/ca9692en.html.

[4] Herforth A , Bai Y, Venkat A, Mahrt K, Ebel A, Masters WA. Cost and affordability of healthy diets across and within countries: background paper for The State of Food Security and Nutrition in the World 2020. FAO Agricultural Development Economics Technical Study No. 9 [Internet]. FAO; 2020; [cited April 25, 2021]. Available from: https://www.fao.org/documents/card/en/c/cb2431en/.

[5] Fanzo J , McLaren R. The product reformulation journey so far: an assessment [Internet]. Geneva: Global Alliance for Improved Nutrition; 2020; [cited April 25, 2021]. Available from: https://www.gainhealth.org/sites/default/files/publications/documents/gain-discussion-paper-series-8-the-product-reformulation-journey-so-far-an-assessment.pdf.

[6] WHO . WHO Fact Sheet: Healthy Diet. [Internet]. Geneva: WHO; 2020; [cited May 4, 2022]. Available from: https://www.who.int/news-room/fact-sheets/detail/healthy-diet.

[7] Weaver C , Dwyer J, Fulgoni VL, King JC, Leveille GA, MacDonald RS et al. Processed foods: contributions to nutrition. Am J Clin Nutr. 2014;99(6):1525–42. 24760975 10.3945/ajcn.114.089284 PMC6410904

[8] Hamilton H , Henry R, Rounsevell M, Moran D, Cossar F, Allen K et al. Exploring global food system shocks, scenarios and outcomes. Futures. 2020;123:102601. 32836328 10.1016/j.futures.2020.102601 PMC7320689

[9] Lehmann U , Mak TN, Bolten CJ. Reformulation as a strategy for developing healthier food products: challenges and recent developments – an industry perspective. In: Raikos V, Ranawana Veditors. Reformulation as a strategy for developing healthier food products. Springer; 2019. p. 89–110.

[10] Mkambula P , Mbuya MNN, Rowe LA, Sablah M, Friesen VM, Chadha M et al. The unfinished agenda for food fortification in low- and middle-income countries: quantifying progress, gaps and potential opportunities. Nutrients. 2020;12(2):354. 10.3390/nu12020354 PMC7071326 32013129

[11] Larousse Gastronomique: The world's greatest culinary encyclopedia. New York (NY): Clarkson Potter/Publishers; 2009.

[12] National Center for Home Food Preservation . USDA complete guide to home canning [Internet]. USDA Publications; 2015; [cited April 25, 2021]. Available from: https://nchfp.uga.edu/publications/publications_usda.html.

[13] Drewnowski A , Almiron-Roig E. Human perceptions and preferences for fat-rich foods. In: Montmayeur JP, le Coutre Jeditors. Fat detection: taste, texture, and post ingestive effects. Boca Raton (FL): CRC Press/Taylor & Francis; 2010. Chapter 11.

[14] Drewnowski A . Taste preferences and food intake. Annu Rev Nutr. 1997;17(1):237–53. 9240927 10.1146/annurev.nutr.17.1.237

[15] Kennedy ET , Buttriss JL, Bureau-Franz I, Klassen Wigger P, Drewnowski A. Future of food: innovating towards sustainable healthy diets. Nutr Bull. 2021;46(3):260–3.

[16] US Food and Drug Administration . Code of Federal Regulations Title 21 CFR 104.20(b) [Internet]. US Department of Health and Human Services; 2020; [cited April 26, 2021]. Available from: https://www.accessdata.fda.gov/scripts/cdrh/cfdocs/cfcfr/cfrsearch.cfm?fr=104.20.

[17] US Food and Drug Administration . Code of Federal Regulations Title 21 CFR 137.165. [Internet]. US Department of Health and Human Services; 2020; [cited April 26, 2021]. Available from: https://www.accessdata.fda.gov/scripts/cdrh/cfdocs/cfcfr/CFRSearch.cfm?fr=137.165.

[18] Global Fortification Data Exchange . Number of food vehicles with selected nutrients in country standards [Internet]. 2021; [cited August 29, 2021]. Available from: https://fortificationdata.org/map-number-of-food-vehicles/.

[19] Prieto-Patron A , Hutton ZV, Fattore G, Sabatier M, Detzel P. Reducing the burden of iron deficiency anemia in Cote D'Ivoire through fortification. J Health Popul Nutr. 2020;39(1):1. 32033590 10.1186/s41043-020-0209-x PMC7006106

[20] Drewnowski A , Garrett GS, Kansagra R, Khan N, Kupka R, Kurpad AV et al. Key considerations for policymakers—iodized salt as a vehicle for iron fortification: current evidence, challenges, and knowledge gaps. J Nutr. 2021;151(Suppl 1):64S–73S. 33582786 10.1093/jn/nxaa377 PMC7882367

[21] Dary O . Avances en el proceso de fortificación de azúcar con vitamina A en Centroamérica [Advances in the process of fortification of sugar with vitamin A in Central America]. Bol Oficina Sanit Panam. 1994;117(6):529–37. 7848541

[22] Mannar V , Diosady L. Quadruple fortification of salt with iodine, iron, vitamins B9 and B12 to reduce maternal and neonatal mortality by reducing anemia and nutritional deficiency prevalence. Curr Dev Nutr. 2019;3(Suppl 1):nzz044

[23] Klassen-Wigger P , Geraets M, Messier MC, Detzel P, Lenoble HP, Barclay DV. Micronutrient fortification of bouillon cubes in Central and West Africa. In: Venkatesh Mannar MG, Hurrell RFeditors. Food fortification in a globalized world. London: Academic Press; 2018. p. 363–72.

[24] Klassen-Wigger P , Barclay DV. Market-driven fortification. In: Venkatesh Mannar MG, Hurrell RFeditors. Food fortification in a globalized world. London: Academic Press; 2018. p. 63–7.

[25] Drewnowski A . Uses of nutrient profiling to address public health needs: from regulation to reformulation. Proc Nutr Soc. 2017;76(3):220–9. 28595659 10.1017/S0029665117000416

[26] Labonté M-È , Poon T, Gladanac B, Ahmed M, Franco-Arellano B, Rayner M et al. Nutrient profile models with applications in government-led nutrition policies aimed at health promotion and non-communicable disease prevention: a systematic review. Adv Nutr. 2018;9(6):741–88. 30462178 10.1093/advances/nmy045 PMC6247226

[27] Storcksdieck genannt Bonsmann S , Marandola D, Ciriolo E, van Bavel R, Wollgast J. Front of pack nutrition labeling schemes: a comprehensive review [Internet]. Publications Office of the European Union; 2020; [cited May 4, 2022]. Available from: https://policycommons.net/artifacts/2162695/front-of-pack-nutrition-labelling-schemes/2918178/.

[28] Gressier M , Sassi F, Frost G. Healthy foods and healthy diets. How government policies can steer food reformulation. Nutrients. 2020;12:1992. 10.3390/nu12071992 PMC7400388 32635525

[29] Combet E , Vlassopoulos A, Molenberg F, Gressier M, Privet L, Wratten C et al. Testing the capacity of a multi-nutrient profiling system to guide food and beverage reformulation: results from five national food composition databases. Nutrients. 2017;9(4):406. 10.3390/nu9040406 PMC5409745 28430118

[30] Access to Nutrition Initiative . Second U.K. product profile 2021 [Internet]. 2021; [cited September 4, 2021]. Available from: https://accesstonutrition.org/news/second-u-k-product-profile-2021/.

[31] Access to Nutrition Initiative . Global index 2021 [Internet]. 2021; [cited September 4, 2021]. Available from: https://accesstonutrition.org/index/global-index-2021/.

[32] Gressier M , Sassi F, Frost G. Contribution of reformulation, product renewal and changes in behavior to the changes in salt intakes of the UK population between 2008-2009 and 2016-2017. Am J Clin Nutr. 2021;114(3):1092–9. 33963735 10.1093/ajcn/nqab130 PMC8408870

[33] Federici C , Detzel P, Petracca F, Dainelli L, Fattore G. The impact of food reformulation on nutrient intakes and health, a systematic review of modelling studies. BMC Nutr. 2019;5:2. 32153917 10.1186/s40795-018-0263-6 PMC7050744

[34] Scrinis G , Monteiro C. Ultra-processed foods and the limits of product reformulation. Public Health Nutr. 2018;21(1):247–52. 28703086 10.1017/S1368980017001392 PMC10261094

[35] Vuik S , Cecchini M. The impact of obesity policies on the food and drink industry. In: The heavy burden of obesity: the economics of prevention. Paris: OECD Publishing; 2019. p. 221–52.

[36] Goryakin Y , Aldea A, Lerouge A, Guillemette Y. Special focus: the health and economic impact of food reformulation. In: The heavy burden of obesity: the economics of prevention. Paris: OECD Publishing; 2019. p. 209–20.

[37] Reardon T , Tschirley D, Liverpool-Tasie SLO, Awokuse T, Fanzo F, Minten B et al. The processed food revolution in African food systems and the double burden of malnutrition. Glob Food Sec. 2021;28:100466. 33868911 10.1016/j.gfs.2020.100466 PMC8049356

